# Monocular Pose Estimation of an Uncooperative Spacecraft Using Convexity Defect Features

**DOI:** 10.3390/s22218541

**Published:** 2022-11-06

**Authors:** Haeyoon Han, Hanik Kim, Hyochoong Bang

**Affiliations:** Department of Aerospace Engineering, Korea Advanced Institute of Science and Technology, 291 Daehak-ro, Yuseong-gu, Daejeon 34141, Korea

**Keywords:** spacecraft pose estimation, vision-based navigation, uncooperative spacecraft, convexity defect

## Abstract

Spacecraft relative pose estimation for an uncooperative spacecraft is challenging because the target spacecraft neither provides sensor information to a chaser spacecraft nor contains markers that assist vision-based navigation. Moreover, the chaser does not have prior pose estimates when initiating the pose estimation. This paper proposes a new monocular pose estimation algorithm that addresses these issues in pose initialization situations for a known but uncooperative target spacecraft. The proposed algorithm finds convexity defect features from a target image and uses them as cues for matching feature points on the image to the points on the known target model. Based on this novel method for model matching, it estimates a pose by solving the PnP problem. Pose estimation simulations are carried out in three test scenarios, and each assesses the estimation accuracy and initialization performance by varying relative attitudes and distances. The simulation results show that the algorithm can estimate the poses of spacecraft models when a solar panel length and the number of solar panels are changed. Furthermore, a scenario considering the surface property of the spacecraft emphasizes that robust feature detection is essential for accurate pose estimation. This algorithm can be used for proximity operations with a known but uncooperative target spacecraft. Specifically, one of the main applications is relative navigation for on-orbit servicing.

## 1. Introduction

Relative navigation in rendezvous, docking, and proximity operations aims to find the accurate relative position and attitude, known as relative pose [1], between a target and a chaser spacecraft [2,3,4]. For cooperative spacecraft, relative navigation using GPS measurement and inter-satellite communication has been widely used in multiple space missions [5,6,7,8]. The studies presented in [9,10,11] introduce technologies relevant to optical communication. Another prominent technology is vision-based relative navigation, which uses vision sensors to estimate accurate relative position and attitude [12]. Previous works [2,3,13,14] for vision-based relative navigation focused on determining the six degrees of freedom relative pose between two spacecraft from an image. The cooperative spacecraft considered in these works have a rhombus-shaped marker [13] or Position Sensing Diode (PSD) sensors [14] on the surface.

Meanwhile, relative navigation for an uncooperative target is of great importance for On-Orbit Servicing (OOS) [15,16] and Active Debris Removal (ADR) [17]. For such missions, vision-based relative navigation can also be used for estimating the pose between an uncooperative target and a chaser [12]. However, uncooperative targets neither employ fiducial markers nor transmit state information. For these reasons, feature point identification is more complicated than the cooperative case [18].

Vision-based approaches are divided into stereo vision and monocular vision, depending on the number of cameras. Stereo vision uses more than two cameras, while monocular vision uses a single camera. Stereo vision can estimate depth using triangulation of the same point that appears in multiple images taken from different viewpoints, but its operational range is limited [19]. On the other hand, although monocular vision has a broader operational range and a faster computational speed, it cannot estimate depth from a single 2D image [20]. As a result, it cannot determine the six degrees of freedom pose because the 3D location of the feature point is unknown within a single image. 

This issue is inherent in monocular vision and has been tackled by employing the shape information of a target [1,4]. Relative pose estimation using monocular vision is divided into model-based and model-free approaches [21]. In the model-based approach, a chaser knows the model of a target in advance. Using the knowledge of the model, it computes the relative pose by mapping points on the target model to points on its 2D projection image [22]. On the other hand, the model-free approach is applied when a chaser does not know the model of a target [21]. In this case, 3D model recovery precedes the pose estimation process. For instance, the 3D point cloud of a target can be acquired by scanning the model with laser radar [23], and the 3D model can be reconstructed by implementing Structure from Motion (SfM) [24]. This paper adopts the model-based approach in which the chaser has a priori information about the target model.

Model-based relative navigation goes through image processing, model matching, pose determination, and pose tracking [24]. In particular, at the beginning of the navigation, there is no prior pose information. This situation is known as pose initialization or pose acquisition [24]. Pose initialization covers from the image processing to pose determination [22], and the following pose tracking uses the initialized pose.

The image processing step, which is the first step of pose initialization, distinguishes the spacecraft from the background to specify the target’s location and then detects the features of the target [22,25,26,27]. Sharma, Ventura, and D’Amico [22] applied Weak Gradient Elimination (WGE) to extract the foreground from the background and detected edges using Hough Transform (HT) [28]. Likewise, Capuano, Alimo, Ho, and Chung [25] eliminated the noise in the image using a Gaussian filter and depicted pixels representing spacecraft using the Gaussian Mixture Model (GMM) [29]. Then, these pixels were processed in parallel by HT, Line Segment Detector (LSD) [30], and Shi-Tomasi corner detection [31] to obtain features.

Next, the model matching step finds mapping from the detected feature points and the points on the target model. This mapping is also referred to as 3D–2D point correspondence [20,22,24]. To find the 3D–2D point correspondences in this step, Capuano, Kim, Harvard, and Chung [24] adopted RANSAC [32]. This algorithm iteratively hypothesizes a match and evaluates the pose error computed from the assumed match to eliminate wrong matches and find the accurate pose [24]. Nonetheless, this method takes a long time to compare the pose error from all random matches [20,22]. As alternatives, for the reduction of search space, Pesce, Opromolla, Sarno, Lavagna, and Grassi [20] used a RANSAC-based approach with Principal Component Analysis (PCA) [33] to determine the distinctive feature points. For the same purpose, Sharma, Ventura, and D’Amico [22] categorized feature points into high-level features, representing figures such as an open polygonal triad and a closed polygonal triad, and found a match from the target points belonging to the same group.

After the model matching step, the pose determination step calculates the pose from the point correspondences [20,22,24]. The pose determination has been handled with a Perspective-n-Point (PnP) algorithm [22,27,34,35,36]. The PnP algorithm finds the relative position and attitude, also known as the camera’s extrinsic parameters, from n-point correspondences between the 2D feature points and known 3D points [37]. Depending on the number of correspondences, the algorithm is categorized into a different algorithm. For instance, a P3P algorithm [38] uses three-point correspondences, and a P4P algorithm [39] uses four-point correspondences to estimate a pose. In addition, the Efficient PnP (EPnP) algorithm [40] produces a pose using greater than or equal to four-point correspondences. In the related studies presented in [27,34], the EPnP algorithm is combined with RANSAC to find a robust pose solution, even if outliers exist in correspondences.

Finally, the pose tracking step continuously estimates the pose from images after the pose is initialized [20,24,36,41]. Capuano, Kim, Harvard, and Chung [24] adopted the SoftPosit [42], and Pesce, Opromolla, Sarno, Lavagna, and Grassi [20] used non-linear filtering techniques for pose tracking. 

More recently, Sharma, et al. [43] adopted Convolutional Neural Networks (CNNs) [44] for pose estimation for detecting robust features from images with a low signal-to-noise ratio and high contrast. Related studies presented in [34,35,36] showed that CNNs can simplify the feature detection and matching process while increasing estimation accuracy. Furthermore, when using CNNs for pose estimation, reducing the domain gap between space imageries and synthetically generated images is another critical topic to be considered [45,46,47]. 

In this study, we aimed to solve the pose initialization problem with uncooperative spacecraft. The key motivation is that accurate pose estimation is critical for rendezvous, docking, and proximity operations of uncooperative spacecraft, but it is difficult to identify the feature points in an acceptable amount of time while achieving high accuracy. In addition, feature point identification becomes more difficult when there is no prior information about the pose between the two spacecraft in the initial stage. 

Earlier works in [20,22,24] also developed pose initialization algorithms, but they have two significant limitations. First, the proposed algorithms in [22,24] are confined to a specific spacecraft model. Pose estimation with the model-based approach exploits a set of reference 3D points on a model, and these points are selected differently for different spacecraft [20]. Although previous works in [22,24] examined the speed and accuracy of the algorithms with the images of a target spacecraft, we cannot be certain that the algorithms are flexible enough to be used for other space missions without verifying the performance with different shapes of spacecraft. 

Second, the performance analyses in [20,22,24] did not consider the effect of the relative pose. The analyses presented in [20,24] described the performance of the pose determination algorithm either with an average time consumed or with pose estimation accuracy under specific scenarios of relative motion. However, the pose determination algorithms depend on the geometry of a target spacecraft, and thus some undesirable relative attitudes could decrease the pose estimation accuracy. For this reason, we cannot evaluate the algorithm’s performance from the average computational time and error. Further analysis of the cases presenting unusual estimation errors was conducted by Sharma, Ventura, and D’Amico [22], but more images are required to comprehensively assess the algorithm’s performance in different relative poses. 

The following summarizes the two main issues this paper tackles.

1. The existing pose estimation algorithms are developed and examined for a specific spacecraft shape.
2. The pose estimation performance analyses often overlook the effect of the relative pose.

This paper proposes a pose estimation algorithm and overcomes these issues. The algorithm detects features from an image, finds 3D–2D point correspondences, calculates a pose, and assesses the reliability of the determined pose in order. The novelty of our work is that it suggests a pose estimation algorithm as an integration of new and existing techniques. The model matching step is newly designed to find 3D–2D point correspondences using convexity defect features. On the other hand, it utilizes earlier works’ ideas for feature detection [48,49] and for pose calculation [38,40]. This paper makes two contributions as follows:

1. We introduce a novel pose initialization algorithm that can apply to target spacecraft with different shapes. This algorithm utilizes a convexity defect to narrow down the search space in the model matching step.
2. The pose determination performance of the algorithm is assessed with various ranges of relative pose and is described by a unique graphical expression of pose error. The pose estimation error is computed for attitudes expressed in azimuth from −180° to 180° and elevation from −90° to 90° while maintaining the relative distance. This process is repeated for five different relative distances.

The rest of the paper consists of five sections. Section 2 defines the pose estimation problem, and Section 3 illustrates essential concepts used for the pose estimation algorithm and a standard spacecraft model. Section 4 elucidates the algorithm in detail, and Section 5 assesses the algorithm using images taken from every viewpoint. Finally, Section 6 concludes the paper. 

## 2. Problem Statement

This paper deals with the pose initialization of an uncooperative spacecraft using monocular vision. Pose estimation in this stage starts without an initial guess, and the images acquired from a monocular camera are the only measurements used to find the pose. However, uncooperative targets neither employ visual markers nor communicate with a chaser spacecraft. Accordingly, we need to find the pose of the target spacecraft solely depending on its natural features. Therefore, the pose estimation problem in this paper is defined as determining the six degrees of freedom pose between the target and chaser spacecraft given an on-board image of the target spacecraft and initializing the pose when the determined pose is reliable.

**Figure 1:**
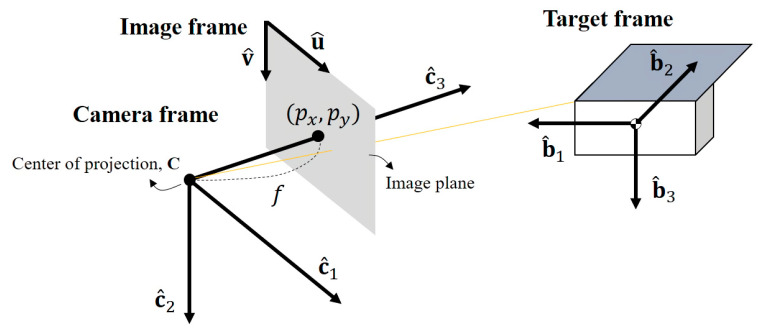
Target frame, camera frame, and image frame in simplified representation.

The pose estimation problem is described in three reference frames in this research as shown in Figure 1. The first is a target frame fixed to its body, and the origin is at its center of mass. The spacecraft’s shape defines the axes of the target frame as shown in Figure 1. For instance, the standard spacecraft model considered in this paper has a cuboid body and a deployed solar panel that extends asymmetrically. With this model, the ${\hat{b}}_{3}$ axis directs the opposite side of the solar panel, the ${\hat{b}}_{2}$ axis is parallel to the direction of the panel extension, and the ${\hat{b}}_{1}$ axis is orthogonal to the ${\hat{b}}_{2}$ and ${\hat{b}}_{3}$ axes. We mark the vector expressed in target coordinates with superscript *T*.

Next, a camera frame is attached to the chaser body, and its origin is at the center of projection, also known as a focal point, where pencils of rays are gathered. The ${\hat{c}}_{3}$ axis points to the image plane from the focal point, and the ${\hat{c}}_{1}$ and ${\hat{c}}_{2}$ axes are parallel to the image plane and head to the right and downward, respectively. A vector expressed in camera coordinates is marked with superscript *C*.

Lastly, an image frame is defined on the image plane. The $\hat{u}$ and $\hat{v}$ axes of the image frame are parallel to the ${\hat{c}}_{1}$ and ${\hat{c}}_{2}$ axes of the camera frame, while the origin is at the corner of the image plane. On this plane, the center of the image plane, or principal point, is at $\left(p_{x},p_{y}\right)$, and a pinhole camera model describes the relationship between the two frames. Here, the focal length *f*, principal point $\left(p_{x},p_{y}\right)$, and pixel size are intrinsic parameters that represent the internal property of the camera, and these parameters are obtained by camera calibration. A vector expressed in image coordinates is marked with superscript *I*. 

The relative pose consists of the relative position and attitude, $t_{C/T}$ and $R_{C/T}$, from the target to the camera, where the target and camera frames are denoted as *T* and *C*, respectively. This paper adopts a model-based approach assuming the chaser has information about the 3D wireframe model of the target in advance. Using this assumption, we determine the relative pose by mapping feature points on the image plane to the known 3D points on the wireframe model of the target. Using the known position of the points on the target expressed in the target frame ${\left(p_{t}\right)}^{T}$, a 3D point on the model can be expressed in the camera frame with the relative position and attitude as follows:

(1) $${\left(p_{t}\right)}^{C}=\left[\begin{array}{c} x_{cam}\\ y_{cam}\\ z_{cam} \end{array}\right]=R_{C/T}\left({\left(p_{t}\right)}^{T}-{\left(t_{C/T}\right)}^{T}\right)$$

Note that the upper-right superscript indicates a reference frame. This 3D point in the camera frame is mapped to a point on the image plane following the pinhole camera model mentioned above. The feature point on the image expressed with the camera coordinates $\left(x_{cam},\;y_{cam},\;z_{cam}\right)$ is depicted by

(2) $${\left(p_{t}\right)}^{I}=\left[\begin{array}{c} x_{img}\\ y_{img} \end{array}\right]=\left[\begin{array}{c} f\frac{x_{cam}}{z_{cam}}+p_{x}\\ f\frac{y_{cam}}{z_{cam}}+p_{y} \end{array}\right]$$

Therefore, a point defined in the target frame can be projected to the image plane through (1) and (2). These equations also suggest that we should know the correct 3D–2D point correspondences to estimate the relative position and attitude.

Briefly, this paper addresses the monocular pose estimation problem of an uncooperative spacecraft without an a priori pose. The pose estimation process starts from finding feature points on the image to matching the feature points with 3D target points and calculating the relative pose from the predicted correspondences. The following sections introduce the detailed method used in this research.

## 3. Concept and Model Description

This section provides an essential concept for pose initialization: a convexity defect. The convexity defect assists pose initialization as a visual cue to identify which point on the model is mapped to the feature point on the image in the model matching step. This step usually takes a RANSAC-based approach combined with an algorithm to select the most probable correspondence candidates among the detected 2D points and 3D target points. The algorithm suggested by this paper also takes the RANSAC-based approach and uses the convexity defect to narrow down the candidates. The following subsections explain the concept of convexity defect and the concepts of a contour and a convex hull required to define the convexity defect. In addition, the standard spacecraft model considered in this paper and assumptions to extract the correspondence candidates on the target are given.

### 3.1. Contour, Convex Hull, and Convexity Defect

For a given image, let us denote the set of feature points on the image plane mapped to the points on the target to $C\subset {\mathbb{R}}^{2}$. The concepts of contour, convex hull, and convexity defect are described within this set C. First, the contour of a set C is a boundary that encloses all points [50]. Some of the points inside the outline do not compose the contour. Next, the convex hull conv C is the smallest convex set that encompasses all points. Its mathematical definition given by Boyd, et al. [51] is written as

(3) $$conv\;C=\{a_{1}x_{1}+\ldots +a_{k}x_{k}\;|\;x_{i}\in C,\;a_{i}\geq 0,\;i=1,\ldots ,\;k,\;a_{1}+\ldots +a_{k}=1\}$$

If a set C is not a convex set, the convex hull is not identical to its contour. In this case, gaps exist between the contour and the convex hull, and the two points that define each gap are considered the start and end points of the convexity defect [52]. The start and end are determined according to the search order of the points in the program. Finally, the point on the contour in the gap and farthest from the convex hull is called a convexity defect [50]. Multiple convexity defects can also exist depending on the contour’s shape. Graham’s scan [53] and Jarvis’s march [54] are the typical methods to acquire the convex hull and the convexity defect. 

**Figure 2:**
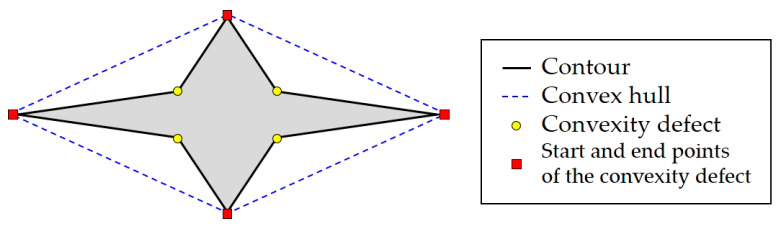
Contour, convex hull, and convexity defect examples.

Figure 2 is an example of representing each concept using a star-shaped object. The black line is the contour, the blue dashed line is a convex hull, the yellow points are the convexity defects, and the red boxes are the start and end points of the convexity defects.

### 3.2. Model Description

**Figure 3:**
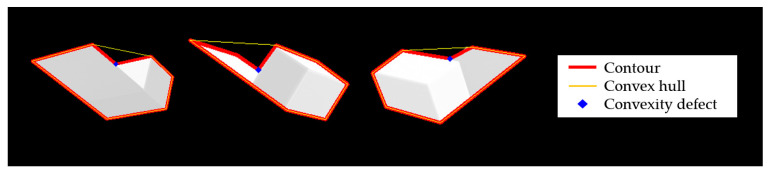
Contour, convex hull, and convexity defect from standard spacecraft model images.

A standard spacecraft model used in this research is a simplified model of a typical spacecraft with a single solar panel on one side. The model replaces the spacecraft’s body and solar panel with a rectangular cylinder and a thin plate. Figure 3 shows the spacecraft model and graphical representations of its contour, convex hull, and convexity defects in three images. Note that the standard spacecraft model has no texture on the surface. The bold red line indicates the contour, the yellow line indicates the edge of the convex hull, and the blue dot indicates the convexity defect. 

**Figure 4:**
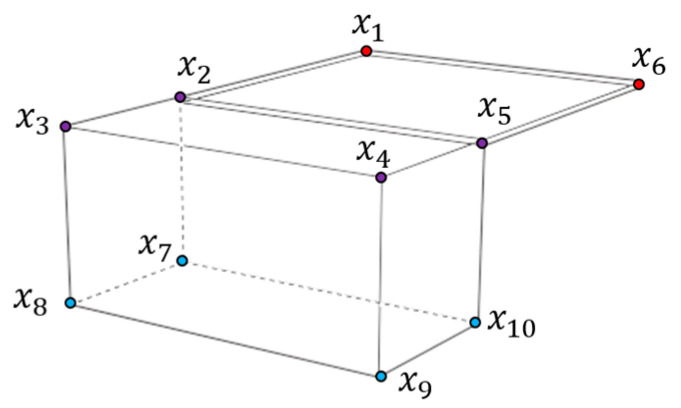
Standard spacecraft model of a spacecraft with a single solar panel.

Figure 4 shows a wireframe model of the spacecraft’s shape with labeled vertices. These vertices are the points we want to detect in the image and can be categorized as a body set B and a panel set P, depending on where they belong. These sets are expressed as

(4) $$P=\left\{x_{1,}x_{2,}x_{3,}x_{4,}x_{5,}x_{6}\right\}\;B=\left\{x_{2,}x_{3,}x_{4,}x_{5,}x_{7,}x_{8,}x_{9,}x_{10}\right\}$$

The intersection of P and B contains the points belonging to the body and the panel, and these points are $x_{2},x_{3},x_{4},$ and $x_{5}$. In contrast, the points belonging to either the body or the panel can be depicted as

(5) $$P\cap B^{c}=\left\{x_{1,}x_{6}\right\}\;P^{c}\cap B=\left\{x_{7,}x_{8,}x_{9,}x_{10}\right\}$$

These classifications are necessary to determine the candidates of 3D points that can be the neighboring points of the convexity defect when the target model is projected to the 2D image plane.

### 3.3. Fundamental Assumptions

The convexity defect is utilized to reduce the search space for finding 3D–2D point correspondences, and it requires four assumptions to select the candidate points on the target. These four assumptions are as follows:

1. If the convex hull and the contour do not coincide, at least one convexity defect exists.
2. The convex hull and the contour become identical if there exist additional lines connecting the points 𝑝 and 𝑏, where $p\in P\cap B^{c}$ and $b\in P^{c}\cap B$
3. Given the simplified model of the spacecraft, the second assumption is further simplified as $p\in P\cap B^{c}=\left\{x_{1},x_{6}\right\}$ and $b\in \left\{x_{7},x_{10}\right\}$.
4. The points p and b determine the start and end points of the convexity defect.

These assumptions effectively rule out the least possible points that map to the start and end points of the convexity defect and thus reduce the search space for finding matches. In addition, we demonstrated these assumptions with images taken from views. The spacecraft model in Figure 3 also follows the assumptions. The first and second images have $p=x_{6}$ and $b=x_{10}$ and the third image has $p=x_{1}$ and $b=x_{7}$.

## 4. Pose Initialization Framework

### 4.1. Overview of Pose Initialization

**Figure 5:**
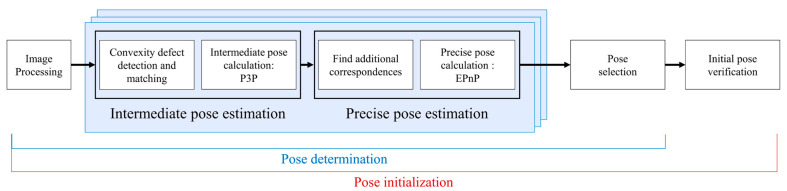
The framework of pose initialization using convexity defects.

The pose initialization algorithm proposed in this paper is composed of five steps, as shown in Figure 5. An image generated from a monocular camera first goes through image processing. Next, the image processing step detects the target’s contour and checks the spacecraft’s location in the image using a bounding box. In the following intermediate pose estimation step, points composing the contour become feature points, and the contour assists in finding the convexity defect. Then, based on the RANSAC algorithm, three points near the convexity defect are chosen and assumed to be mapped to the predetermined 3D points on the target. This step produces multiple correspondence sets between an image and the standard model, which lead to multiple intermediate pose solutions computed from the P3P algorithm. In the third step, each intermediate pose solution is exploited to find additional correspondences, and a more precise pose is calculated by applying the EPnP algorithm. The precise pose solutions are examined through error metrics, and the pose with the minimum error is chosen as the image’s final pose. Finally, to make the initial pose reliable, the pose initialization terminates when the reprojection error of the final pose is smaller than the predetermined threshold.

We refer to the procedure from the image processing step to the initial pose verification step as pose initialization and the procedure from the image processing step to the pose selection step as pose determination. The pose initialization algorithm is given in Algorithm 1. This algorithm comprises sub-algorithms for each step, and the details of the sub-algorithms are presented in the following subsections.
**Algorithm 1:** Pose initialization algorithm  **Input:** Image **sub-algorithm** Image processing (Algorithm 2) **if** the contour is detected and nonconvex **then**         **sub-algorithm** Intermediate pose estimation (Algorithm 3) **sub-algorithm** Precise pose estimation (Algorithm 4) **sub-algorithm** Pose selection (Algorithm 5) **sub-algorithm** Initial pose verification (Algorithm 6)     **else**     Go back to the beginning and read another image **end**

**Algorithm 2:** Sub-algorithm for the image processing step  Blur the Image Binarize the blurred image Extract the contour from the binary image Compute the bounding box from the binary image **if** the contour is detected **then**             Get the simplified contour from the detected contour Extract vertices from the simplified contour   
**else**
     Go back to the beginning and read another image 
**end**


**Algorithm 3:** Sub-algorithm for the intermediate pose estimation step  Extract a convexity defect and the start and end points of the convexity defect Check that the contour corresponds to case 1 or case 2  *comb_2d* = a set of 2D point combinations *comb_3d* = a set of 3D point combinations **for** *num_2d* = 1 **to** the number of triads in *comb_2d*             **for**
*num_3d* = 1 **to** the number of triads in *comb_3d*          *corr_set* = correspondence between *comb_2d*[*num_2d*] and *comb_3d*[*num_3d*] Compute intermediate poses using the P3P algorithm Add the intermediate poses to the intermediate pose set, *int_pose*    
**end**
 
**end**


**Algorithm 4:** Sub-algorithm for the precise pose estimation step  **for** *num_pos* = 1 **to** the number of poses in *int_pose*     Project the 3D points to an image plane using *int_pose*[*num_pos*] **for**
*i* = 1 **to** the number of total 3D points       **for** *j* = 1 **to** the number of extracted feature points      Compute $r_{ij}$ 
**if**
$r_{ij}\leq r_{\mathrm{ref}}$
**then**      Add the *i*th 3D point and the *j*th 2D point to *corr_set*
**end**

**end**
  
**end**
   **if** the number of correspondences in *corr_set* > 3 **then**       Compute a precise pose using the EPnP algorithm Add the precise pose to the precise pose set, *prec_pose*    
**end**
 
**end**


**Algorithm 5:** Sub-algorithm for the pose selection step
 **for** *l* = 1 **to** the number of elements in *prec_pose*     Project the 3D points to an image plane using *prec_pose*[*l*] Find the bounding box of the reprojected 3D points Compute IOU **if** IOU > 0.8 **then**          Compute $r_{\mathrm{total},l}$ 
**if**
$r_{\mathrm{total},l}$$\;<\;r_{{\mathrm{total}}_{\min}}$
**then**             *pose_solution* = *prec_pose*[*l*] $r_{{\mathrm{total}}_{\min}}$$=r_{\mathrm{total},l}$
      
**end**
    
**end**
 
**end**


**Algorithm 6:** Sub-algorithm for the initial pose verification step  **if** $r_{{\mathrm{total}}_{\min}}$
$<\;r_{{\mathrm{total}}_{\mathrm{thd}}}$
**then**     *initial_pose* = *pose_solution* **return** *initial_pose* (end of pose initialization) 
**else**
     Go back to the beginning and read another image 
**end**


### 4.2. Image Processing

Image processing aims to obtain features suitable to calculate the pose in the later steps. We use OpenCV library functions [55] to detect the target’s contour in the image and extract feature points from the contour.

The sub-algorithm for the image processing step is given in Algorithm 2. First, a Gaussian filter applies to the raw image to blur it. The blurred image helps ignore the surface texture that might induce the detector to find undesirable points. Second, image binarization is used to detect the edges and points on the target. Although binarization omits color and brightness information, the binarized image is suitable for extracting the shape of the target in the image. Third, the bounding box and the contour are detected.

**Figure 6:**
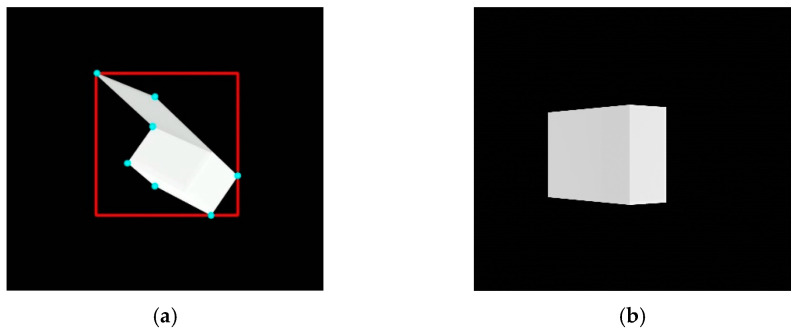
Image processing results: (**a**) bounding box and feature points represented by a red box and cyan dots and (**b**) target image with a convex contour.

The algorithm chooses feature points from the detected contour. However, the contour usually contains successive points along the contour line, while the algorithm requires a few points discriminable from the other points. Thus, the corners of the contour are selected as feature points. Then, the algorithm checks whether the simplified contour composed of the selected feature points is convex. If the simplified contour is convex, the algorithm stops finding the relative pose and starts from the beginning of image processing with the next image. The reason is that this algorithm cannot find 3D–2D point correspondences if the convexity defect does not exist. On the other hand, if the simplified contour is nonconvex–which means that there is at least one convexity defect–the algorithm moves on to the subsequent process. Figure 6 depicts the bounding box and feature points for a nonconvex contour and pose estimation failure image with a convex contour.

### 4.3. Intermediate Pose Estimation

The intermediate pose estimation step receives detected features as input and generates intermediate pose estimates. An intermediate pose in this paper refers to a low-accuracy pose necessary to find a more precise pose. In this step, the detected features go through model matching and pose determination to compute an intermediate pose from the features.

The algorithm proposed in this paper is based on RANSAC, and a convexity defect provides a clue for identifying the 2D projection of 3D points with fewer iterations. The algorithm assumes group correspondences by constructing 3D and 2D point combinations. Then, it employs the P3P algorithm to estimate an intermediate pose using the correspondences. Since the P3P algorithm requires three correspondences, the 2D and 3D point combinations have three elements.

Algorithm 3 shows the sub-algorithm for the intermediate pose estimation step. First, to construct a 2D point combination, a convexity defect and its start and end points are detected from the simplified contour. Then, two 2D points are selected from the start and the end points of the convexity defect, and the other 2D point is selected from the point near the start or end point.

Second, this 2D point combination is assumed to correspond to one of the 3D point combinations. The 3D point combinations are predetermined before the algorithm runs. Based on the assumptions introduced in Section 3.3, two candidate 3D points corresponding to the start and end points of the convexity defect are assumed. In addition, a 3D point corresponding to the other 2D point is assumed by considering the model’s geometry. The 2D and 3D point combinations used in this paper are given in Table 1. 

**Figure 7:**
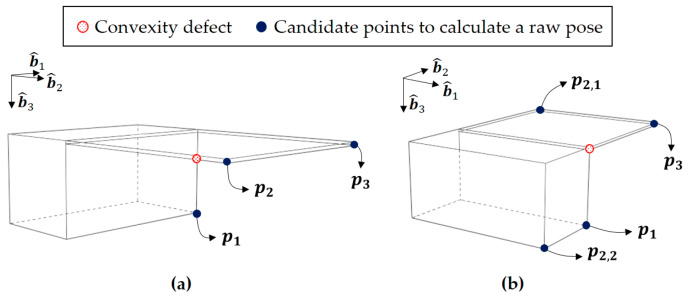
Candidate feature points in two cases. (**a**) case 1, (**b**) case 2.

When constructing the 2D point combination, the neighboring point of the start or end point is determined by the number of detected corners between the start point and end point, as shown in Figure 7. In this figure, the start and end points of the convexity defect are designated as $p_{1}$ and $p_{3}$. If there are more than two points between them, which is case 1, the point not a convexity defect becomes $p_{2}$ and completes the feature point combination. Otherwise, in case 2, the neighboring point of the start point or end point, which is $p_{2,1}$ or $p_{2,2}$, is selected to consider all possibilities. The number of feature point combinations is one in case 1 since $p_{2}$ is obvious, whereas the number of combinations in case 2 is two since we cannot predict which neighboring point will provide a more precise solution. 

Finally, after assuming the 3D–2D point correspondences, intermediate poses are calculated using the P3P algorithm. It gives four candidate poses at maximum, and each pose is separately processed until their errors are compared to each other to rule out false ones in the pose selection step. Section 4.5 provides more explanation for pose selection and error metrics.

### 4.4. Precise Pose Estimation

The precise pose estimation step is designed to refine the intermediate pose. The intermediate pose calculated in the previous step expands the 3D–2D point correspondences to acquire a more precise pose. The target points are projected to the image plane using the intrinsic parameters of the camera and the extrinsic parameters obtained from the intermediate pose. This method is also known as reprojection, and the points on the image generated from reprojection are considered reprojected points. Using these points, we can define a reprojection error as the distance between a reprojected point and the nearest feature point. It is given by,

(6) $$r_{ij}=\sqrt{{\left(u_{3D,i}-u_{img,j}\right)}^{2}+{\left(v_{3D,i}-v_{img,j}\right)}^{2}},\;i=1,2,\ldots ,n,j=1,2,\ldots ,m$$

where $u_{3D,i}$ and $v_{3D,i}$ represent the coordinates of the *i*th reprojected point, $u_{img,j}$ and $v_{img,j}$ are the coordinates of the *j*th feature point, and n and m are the total number of target points and feature points, respectively. If the intermediate pose is accurate, some reprojected points coincide with the feature points–except the occluded ones. This situation can be expressed as

(7) $$r_{ij}=0$$

Otherwise, if the reprojected point and the feature point are in correspondence but do not coincide, the reprojection error has a value less than or equal to the reference value, $r_{\mathrm{ref}}$:

(8) $$r_{ij}\leq r_{\mathrm{ref}}$$

The 3D and 2D points combination is added to the existing 3D–2D point correspondences in this case.

If the reprojection error is greater than the reference value, the two points are considered different:

(9) $$r_{ij}>r_{\mathrm{ref}}$$

**Figure 8:**
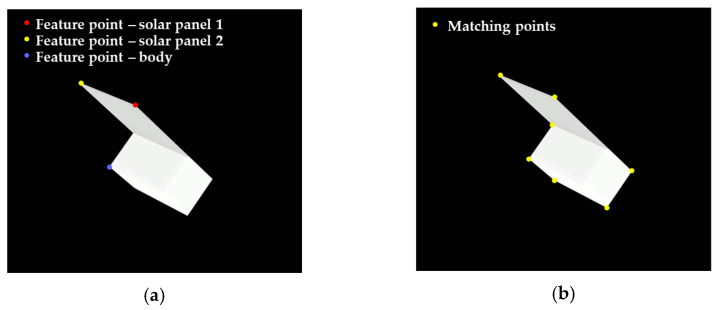
Feature points for (**a**) intermediate pose estimation and (**b**) precise pose estimation.

Figure 8 shows the feature points having a match before and after expanding the correspondences.

If the above process finds more than one correspondence, more than four pairs of 3D–2D point correspondences are known. These correspondences are used for precise pose estimation. The EPnP, which gives a more accurate solution than the P3P by using more correspondences, is employed this time since the requirement on the number of correspondences is now satisfied. In this way, each hypothesized correspondence determines a precise pose. The sub-algorithm for precise pose estimation is described in Algorithm 4.

### 4.5. Pose Selection

So far, the poses are estimated from the candidate correspondences to consider possibilities. The pose selection step determines the best estimation of the pose using two criteria. The first one is a bounding box similarity. To compare the bounding box similarity, we use a precise pose estimate to reproject the target points onto the image plane and compute the reprojected bounding box from these points. The similarity between the reprojected bounding box and the feature point bounding box is determined using Intersection Over Union (IOU), which is frequently used as a performance measure in object detection problems [56]. IOU represents the similarity as an overlapping percentage, which is depicted by

(10) $$\mathrm{IOU}=\frac{\left(\mathrm{Area}\;\mathrm{of}\;\mathrm{the}\;\mathrm{intersection}\right)}{\left(\mathrm{Area}\;\mathrm{of}\;\mathrm{the}\;\mathrm{union}\right)}$$

This criterion rules out the pose estimates when reprojected points significantly deviate from the bounding box computed from the feature points. In Section 5, the pose estimates with IOU less than 0.8 are regarded as inaccurate and are rejected in the final pose candidates.

The pose estimates that satisfy the bounding box similarity criterion are examined for the second criterion, a sum of reprojection errors. The formulation for a reprojection error applies the same as in the previous step. However, in this step, we add the errors from *i* = 1 to *i* = *k* correspondences to compare with other estimates’ errors. The sum of the reprojection error is given by

(11) $$r_{\mathrm{total}\;=}\overset{k}{\underset{i=1}{\sum }}\sqrt{{\left(u_{3D,i}-u_{img,i}\right)}^{2}+{\left(v_{3D,i}-v_{img,i}\right)}^{2}},\;k<n\;$$

The reason for using k points is that the reprojection error might have a considerable value when some of the points are occluded, even though the estimation is accurate.

**Figure 9:**
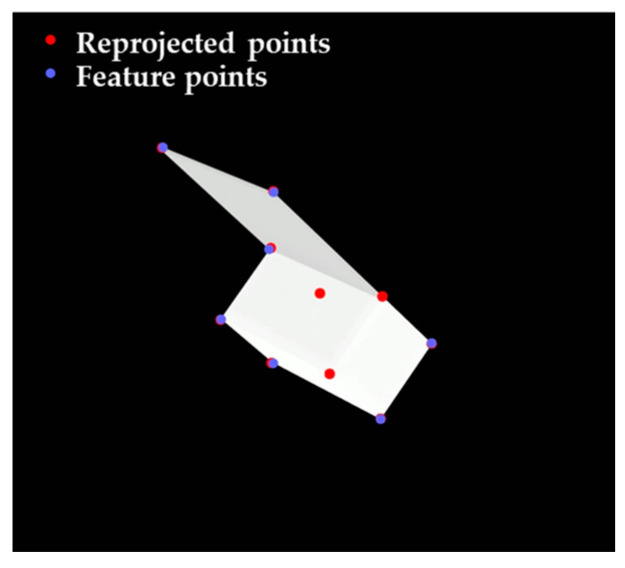
Reprojected points and feature points.

For example, Figure 9, describing the reprojected points and feature points, indicates that some reprojected points do not match when they are not at the corner of the contour. Therefore, considering the possible occlusions, k points instead of the total number of target points are used for calculating the sum of reprojection errors, and the points to be used are selected in the order of smallest reprojection error. The number of selected points can differ according to the camera’s angle and the target’s shape, and we use five points, half of the total target points. Finally, the estimated pose with the smallest sum of reprojection errors is determined as the final pose for the given image. Algorithm 5 shows the sub-algorithm for the pose selection step.

### 4.6. Initial Pose Verification

The last step of pose initialization is to examine whether the finalized pose is accurate. This step is necessary to find a reliable initial pose in the pose initialization step and to move on to the pose tracking. Algorithm 6 describes the sub-algorithm for the initial pose verification step. The decision is made from the sum of the reprojection errors that effectively represents the estimation quality. If the sum of the reprojection errors computed at the previous step is smaller than a threshold, $r_{{\mathrm{total}}_{\mathrm{thd}}}$, the determined pose is assumed to be accurate, and the pose initialization is finished. On the other hand, if the determined pose has a reprojection error larger than the threshold, the pose initialization steps are repeated with the next image.

## 5. Simulations

### 5.1. Simulation Environments and Performance Measures

Pose estimation simulations are conducted to examine the performance and analyze the characteristics of the proposed algorithm. The simulations use image data generated by 3D software, Blender [57], with the camera setting given in Table 2. The camera setting is determined by referring to the Digital Video System (DVS) used for the PRISMA mission [58]. Since the simulations aim to analyze the performance depending on distances and attitudes, the image data for the simulations are generated by rotating the camera around a target spacecraft model. The camera is at (0, ρ, 0) in the target coordinates at the beginning, where ρ represents the distance from the target to the camera. Then, it moves 10° per each axis: from 0° to 360° in ${\hat{b}}_{3}$ direction, from −90° to 90° in ${\hat{b}}_{1}$ direction, and from 0° to 360° in ${\hat{b}}_{2}$ direction with the rotation sequence of 3-1-2. In this way, 22,104 images are generated for each test case.

As mentioned in Section 3, the standard spacecraft model is designed to represent a spacecraft with one solar panel on a side, and its dimensions used in the simulations are described in Table 3. For the given dimensions, the pose estimation with the camera specifications in Table 2 shows relatively accurate results within 20 m to 75 m distance.

We use the apparent angular size to describe the degree of proximity between the spacecraft instead of the distance between them. When a specific target spacecraft for a mission is determined, only the distance between the target spacecraft and the chaser spacecraft affects the size of the target in an image because the dimensions of the target spacecraft have fixed values. However, the standard spacecraft model used in this paper does not represent a specific target spacecraft but a typical spacecraft with a single solar panel, and it can have dimensions different from the values given in Table 3; accordingly, the target’s size in an image can also change, even if the distance between the spacecraft is the same. Therefore, we adopt apparent angular size to consider that the model’s dimensions might change.

We use the apparent angular size from Woffinden and Geller [59], in which both angles-only navigation and pose estimation for rendezvous missions have been studied. This study modeled a target as a bounding sphere that shares the centroid with the target. Using the known diameter of this sphere $D_{target}$ and the apparent angular size $\theta_{target}$ in an image plane, the relative distance ρ between them can be depicted by

(12) $$\rho =\frac{D_{target}}{\theta_{target}}$$

As (12) suggests, the relative distance and the target scale influence each other. Thus, we set the apparent angular size $\theta_{target}$, which practically affects the pose estimation performance, as a metric that shows the degree of proximity within the same spacecraft model. This metric is expressed as

(13) $$\theta_{target}=\frac{D_{target}}{\rho }$$

The performance of the algorithm is analyzed with four measures. A translation error and an attitude error represent the performance of the pose determination. We follow the definitions in Sharma and D’Amico [60], which are given by

(14) $$E_{T}=\frac{\left|t^{C}|-|{\hat{t}}^{C}\right|}{\left|t^{C}\right|}\cdot 100\;[\%]$$

(15) $$E_{R}=2\cos^{-1}q_{e,4}$$

where

(16) $${\bar{q}}_{e}=\bar{q}⊗{\hat{\bar{q}}}^{-1}=\left[\begin{array}{c} q_{e}\\ q_{e,4} \end{array}\right]$$

The other two measures are the pass rate and the outlier ratio, describing the performance of the pose initialization algorithm given in Algorithm 1. The pass rate represents the ratio of images that passes the pose initialization algorithm among all images taken from the same relative distance. It is depicted by

(17) $$\frac{n_{pass}}{n_{tot}}\cdot 100\cdot [\%]$$

where $n_{tot}$ is the number of test cases that have the same relative distance, and $n_{pass}$ is the number of test cases that pass pose initialization. 

Finally, the outlier ratio shows the percentage of faulty poses that passes the sub-algorithm for initial pose verification described in Algorithm 6. It is expressed as

(18) $$\frac{n_{out}}{n_{pass}}\cdot 100\cdot [\%]$$

where $n_{out}$ is the number of faulty poses. When using this ratio as a performance measure, the pose estimation result with more than 5% position error or more than 10° attitude error is assumed to be the outlier. 

The simulation is conducted based on four assumptions. First, the target spacecraft is always in the image, even if some parts are out of view when the relative distance is short. Secondly, any other celestial bodies and the Earth do not appear in the image; thus, the image’s background is uniformly black. Thirdly, the light source is fixed to one location when generating images. Finally, images have no distortion, and the camera’s intrinsic parameters are known in advance.

### 5.2. Algorithm Effectiveness Assessment

Before analyzing the performance of the proposed pose estimation algorithm, a preliminary simulation is designed to evaluate the effectiveness of the proposed algorithm. This simulation is subdivided into two simulations. The first sub-simulation assesses the effectiveness of model matching by comparing our algorithm’s results to that of the RANSAC algorithm not employing visual cues. The second sub-simulation assesses the effectiveness of our algorithm’s structure. For this sub-simulation, a simplified algorithm is designed to analyze the effectiveness of the integration.

#### 5.2.1. Effectiveness Assessment of Model Matching

Not using visual cues, the RANSAC algorithm compares all combinations of 3D and 2D points in the first sub-simulation. This algorithm randomly selects four points each from the given feature points and the known 3D points on the target model and assumes a correspondence between them. Then, based on this correspondence, the EPnP algorithm calculates a pose. Since the RANSAC algorithm uses the EPnP algorithm, we use four correspondences, which is the minimum number of correspondences required. Finally, when a pose has an IOU higher than 0.85 and the minimum reprojection error, it is selected as the pose for the given image. Briefly, the intermediate pose estimation step is removed from the proposed algorithm, and the precise pose estimation step considers all correspondences.

The proposed pose estimation algorithm and the RANSAC algorithm are tested for all viewpoints and the same apparent angular size of 15.7° corresponding to the relative distance of 30 m. The accuracy of the pose determination is computed using (14) and (15). However, if an algorithm fails to produce a pose from the given image, the relative pose error is expressed with a threshold value for the pose error. We assume 10% for the position error threshold and 100° for the attitude error threshold.

**Figure 10:**
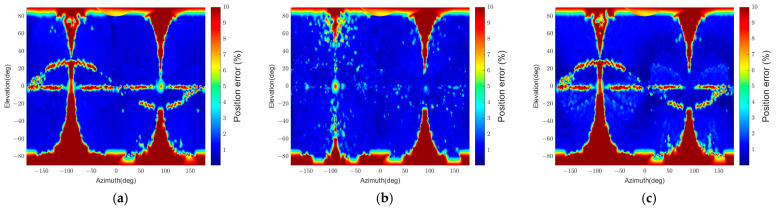
Relative position errors for different algorithms ($\theta_{target}=15.7{}^{\circ}$): (**a**) CDA, (**b**) RANSAC, (**c**) CDA-simple.

**Figure 11:**
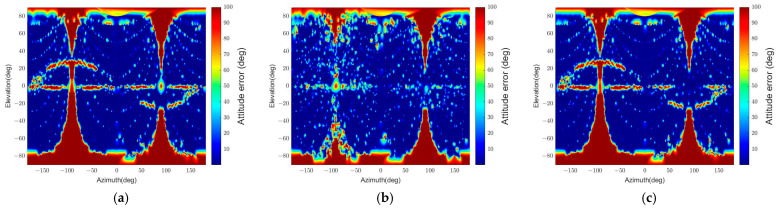
Relative attitude errors for different algorithms ($\theta_{target}=15.7{}^{\circ}$): (**a**) CDA, (**b**) RANSAC, (**c**) CDA-simple.

The resulting position and attitude errors from the two algorithms are shown in Figure 10 and Figure 11, respectively. “Convexity Defect-based Algorithm (CDA)” denotes our algorithm, and “RANSAC” indicates the RANSAC algorithm. The graphical representations are generated by interpolating the pose errors at all viewpoints and plotting them on the 2D plane using the azimuth and elevation. As the error increases from 0 to the threshold value, the color changes from blue to red. 

Figure 10 indicates that the RANSAC algorithm has a low pose determination error in most viewpoints. However, there are regions with the maximum position error. The maximum position error appears because the target’s panel occludes the body, and thus a small number of points on the same plane are detected. This results in a low estimation accuracy since the EPnP algorithm’s accuracy drops when detected points are coplanar in 3D, and the number of points is less than 5 [61,62]. In addition, in Figure 11, the relative attitude error of RANSAC is inaccurate in more regions than the relative position error in Figure 10 because of pose ambiguity. Compared to RANSAC, CDA shows inaccurate results in more regions, as shown in Figure 10 and Figure 11. It also has a particular error pattern that appears when it fails to detect convexity defects. A more specific analysis of the pattern is presented in Section 5.3.1.

Table 4 summarizes the estimation results. The time in the table is the execution time when the algorithm runs on an Intel Core i7-10700 CPU @ 2.90 GHz with 16 GB RAM. The relative position and relative attitude in the table represent the statistical values of relative position and attitude errors. The statistic values consider the errors of poses that passed the pose verification step, and outliers are excluded. As the pose determination results in Figure 10 and Figure 11 reveal, the pass rate for RANSAC is higher than for CDA, but the outlier ratio is also higher.

Furthermore, RANSAC’s errors are similar to or worse than CDA’s since it uses only four points in the pose calculation. If RANSAC uses more than four points to improve its accuracy, the execution time will increase. In contrast, CDA requires about 50 times less execution time than RANSAC while providing higher relative attitude accuracy and similar relative position accuracy. Thus, the results indicate that CDA is an effective and time-efficient algorithm.

#### 5.2.2. Effectiveness Assessment of Algorithm’s Structure

In the second sub-simulation, the effectiveness of the Convexity Defect-based algorithm’s structure is verified by comparing it to a more simplified algorithm. The simplified algorithm does not have the precise pose estimation step. Accordingly, the pose is determined to be one of the solutions of the P3P algorithm with a minimum reprojection error.

The simplified algorithm is denoted “CDA-simple.” Figure 10 and Figure 11 reveal that the pose determination accuracy for CDA and CDA-simple is similar and has a similar error distribution. A minor difference is that the CDA has a more homogeneous position error distribution than the CDA-simple, as shown in Figure 10.

Table 4 explains why the precise pose estimation step, included only in CDA, is necessary. The total execution time difference between the two algorithms is 753 s. Considering that one set of simulations comprises 22,104 images, CDA takes about 0.034 s per image more than CDA-simple by including the precise pose estimation step. By compromising this time, CDA can achieve a more accurate pose estimation. The pass rate increases by 0.11% in CDA and has a lower outlier ratio. In addition, the statistical errors for relative position and attitude reveal that CDA has a lower mean and standard deviation of errors. Hence, the second sub-simulation shows that CDA can enhance pose estimation accuracy without sacrificing computational efficiency as much as RANSAC. 

### 5.3. Simulation Scenarios for Performance Analysis

**Figure 12:**
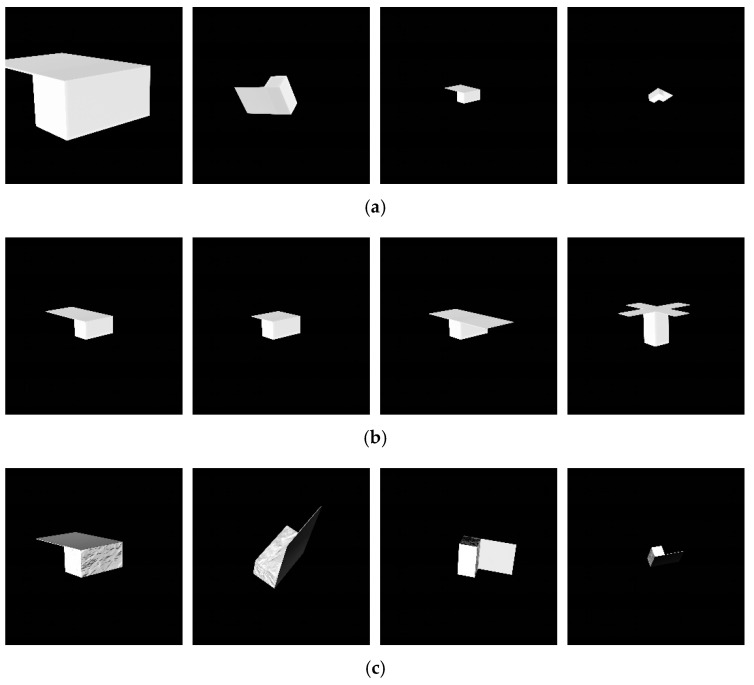
Example images for each scenario: (**a**) scenario 1 (images with different relative poses), (**b**) scenario 2 (images with different lengths and numbers of the panel), and (**c**) scenario 3 (images with textured-surface spacecraft).

After the preliminary simulation, three more simulations are designed to test the pose initialization performance of our algorithm in different conditions. In the first scenario, the performance is examined under five apparent angular sizes, from 47.1° to 6.3°, and all viewpoints. The second scenario tests the pose initialization and determination performances using other spacecraft shapes. This scenario uses spacecraft models with a panel shorter and longer than the standard model’s panel and models with two and four panels. Finally, the algorithm is tested using a textured spacecraft model that generates high-contrast images depending on the direction of light in the last scenario. The model’s body is covered with MLI, and the solar panel is covered with a black reflective material. Images representing the scenarios are given in Figure 12. 

#### 5.3.1. Pose Estimation Performance Depending on Relative Poses

The first test scenario analyzes our algorithm’s performance depending on relative poses. It uses image data from five different relative distances. The tested apparent angular sizes are 47.1°, 23.5°, 15.7°, 9.4°, and 6.3°, and they correspond to the relative distances of 10 m, 20 m, 30 m, 50 m, and 75 m for the spacecraft with the scale given in Table 3.

**Figure 13:**
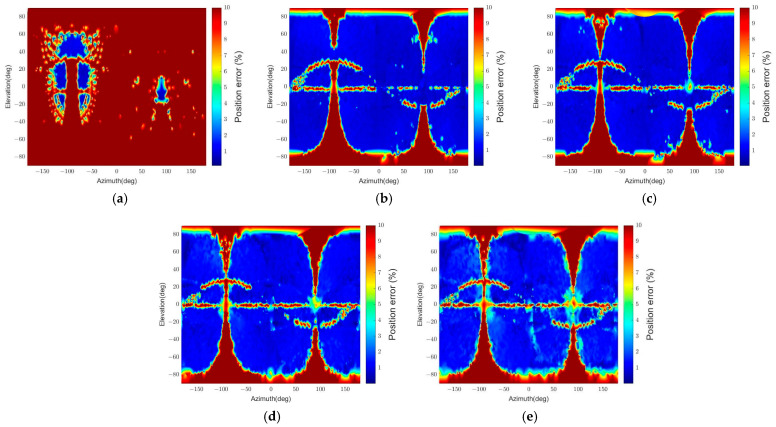
Relative position error for different relative poses. The position errors over 10% are integrated into the maximum value of 10%. (**a**) $\theta_{target}=47.1{}^{\circ}$, (**b**) $\theta_{target}=23.5{}^{\circ}$, (**c**) $\theta_{target}=15.7{}^{\circ}$, (**d**) $\theta_{target}=9.4{}^{\circ}$, (**e**) $\theta_{target}=6.3{}^{\circ}$.

**Figure 14:**
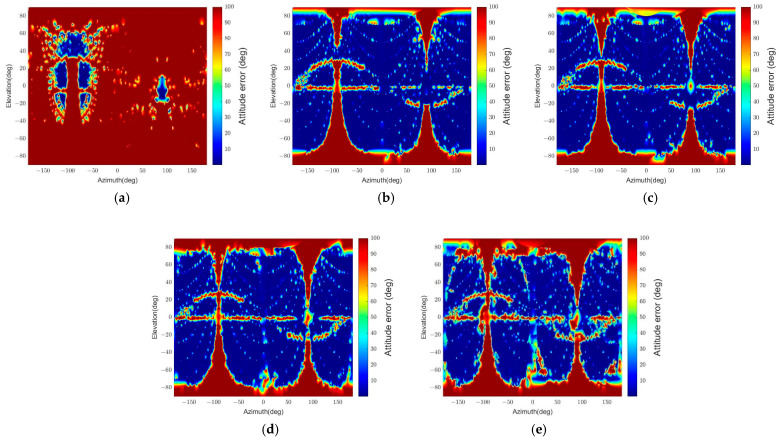
Relative attitude errors for different relative poses. The attitude errors over 100° are integrated to the maximum value of 100°. (**a**) $\theta_{target}=47.1{}^{\circ}$, (**b**) $\theta_{target}=23.5{}^{\circ}$, (**c**) $\theta_{target}=15.7{}^{\circ}$, (**d**) $\theta_{target}=9.4{}^{\circ}$, (**e**) $\theta_{target}=6.3{}^{\circ}$.

We can notice the effect of apparent angular size on the pose determination accuracy in Figure 13 and Figure 14. The error grows as the angular size decreases due to the low pixel resolution. In addition, the smaller the angular size, the more challenging it is for the algorithm to distinguish the different orientations with 2D points in similar locations when projected. This result appears because the algorithm uses the points on the contour, and the standard spacecraft model has symmetry. Moreover, the sum of reprojection errors, which reflects a few points’ reprojection errors, makes it difficult to determine an accurate pose.

In addition, the error also escalates if the two spacecraft are so close that the camera’s field of view cannot capture every part of the target. More specifically, when the target has an apparent angular size larger than about double the field of view, some parts are not shown in the image, which makes feature point detection difficult. Further, the parts far from the center can also be invisible when the target spacecraft appears at the periphery of the image. Therefore, the distance between the spacecraft and the line of sight are essential factors that affect the pose determination performance.

**Figure 15:**
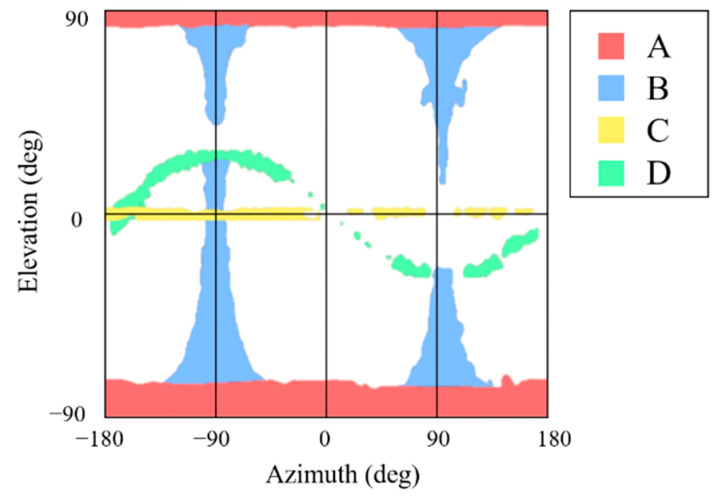
Pose determination failure parts with significant errors. A: ±90° of elevation region, B: ±90° of azimuth region, C: zero elevation region, D: the sinusoidal region in the diagram.

**Figure 16:**
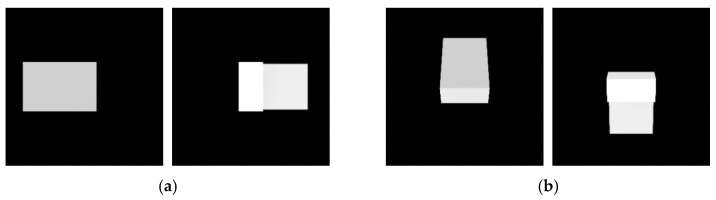

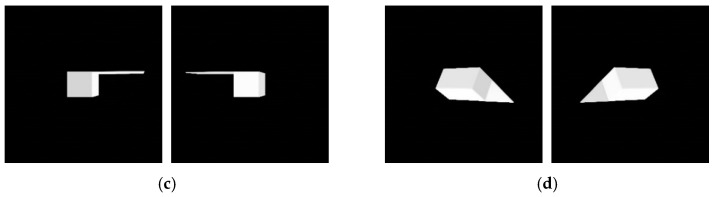
Example images for failure sections: (**a**) failure section A, (**b**) failure section B, (**c**) failure section C, (**d**) failure section D.

Furthermore, the relative position and attitude errors in Figure 13 and Figure 14 show that some regions have threshold values for position and attitude errors, which indicates that the poses are not determined in these regions. These regions are categorized into four parts according to the causes of pose determination failure, as shown in Figure 15. Firstly, the error increases near ±90° of elevation marked ‘A’ in Figure 15. In this part, the ${\hat{b}}_{1}$ and ${\hat{b}}_{2}$ axes of the target spacecraft are nearly parallel to the image plane, and it is difficult to distinguish the body and the panel. Secondly, the error grows near ±90° of azimuth marked ‘B’ since the target’s contour becomes convex and the line connecting p and b in the assumptions presented in Section 3.3 is always inside or overlaps with the contour. The third part, ‘C,’ is where the elevation is zero. In this part, the solar panel is nearly perpendicular to the image plane and projected to a line. Lastly, a sinusoidal region is discovered in Figure 13 and Figure 14 and marked ‘D’ in Figure 15. The convexity defect is also invisible in this part because a plane composed of the edge of the solar panel $\bar{x_{1}x_{6}}$ and the focal point contains the edge of the body $\bar{x_{7}x_{10}}$. As a result, the two edges look like a connected line in the image. These failure parts indicate that the pose determination accuracy deteriorates when the convexity defect does not appear in the target image. Moreover, it indicates which viewpoint fails to find the convexity defect. Figure 16 shows example images from the failure parts.

Next, the pose initialization performance is checked with the pass rate and the outlier ratio and analyzed according to apparent angular size. The pose initialization algorithm has a final step to verify the estimated pose, and the pose with less than a reprojection error criterion can pass this step. The reprojection error criterion is assumed to be 400 divided by the five relative distances to reflect a change of reprojection error. However, if this criterion is expressed in a pixel unit, it filters out more poses when the relative distance is shorter. With this distance-dependent criterion, the pass rate and the outlier ratio describe the pose initialization performance according to relative poses.

The first measure, the pass rate, indicates the ratio of getting a good pose solution that passes the sub-algorithm for the initial pose verification step given in Algorithm 6. As Table 5 describes, the pass rate drops when the inter-satellite distance is too short to picture the whole spacecraft or too far to recognize it in high resolution. These results are consistent with the pose determination results. 

The second measure is the number of outliers. Table 5 shows the percentages of outliers among the initialized poses. For the analysis, the estimated pose with more than 10° of relative attitude error or more than 5% of position error is regarded as an outlier. These values are much larger than three standard deviations of the mean for total errors, including outliers’ errors. With these conditions, Table 5 implies that outliers increase when the inter-satellite distance is extremely short or extremely far. 

Finally, Table 6 shows the relative pose solutions without outliers depending on apparent angular size. In this table, both the position and attitude errors have a larger mean and standard deviation as the chaser spacecraft is farther apart from the target spacecraft. The mean values for these results without outliers are smaller than 1.1° for the relative attitude and 1.4% for the position.

#### 5.3.2. Pose Estimation Performance Depending on the Shape of a Spacecraft

The third scenario is designed to verify whether the proposed algorithm can accurately estimate the relative pose of a spacecraft that is different from the standard spacecraft used for developing the algorithm. Specifically, this scenario analyzes the effect of the panel length on the pose estimation performance and the effect of the number of panels on the performance. First, the algorithm is applied to spacecraft with different sizes of solar panels. The panel lengths are expressed as the width ratio between the solar panel and the body to generate the value independent of the spacecraft’s size. The width ratio is expressed as

(19) $$W_{\mathrm{bp}}=\frac{\left(\mathrm{Width}\;\mathrm{of}\;\mathrm{the}\;\mathrm{panel}\right)}{\left(\mathrm{Width}\;\mathrm{of}\;\mathrm{the}\;\mathrm{body}\right)}$$

**Figure 17:**
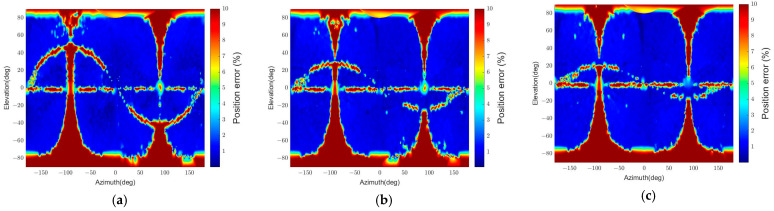
Relative position errors with variable size solar panels ($\left|r\right|$ = 30 m): (**a**) $W_{\mathrm{bp}}=1$, (**b**) $W_{\mathrm{bp}}=2$, (**c**) $W_{\mathrm{bp}}=3$.

**Figure 18:**
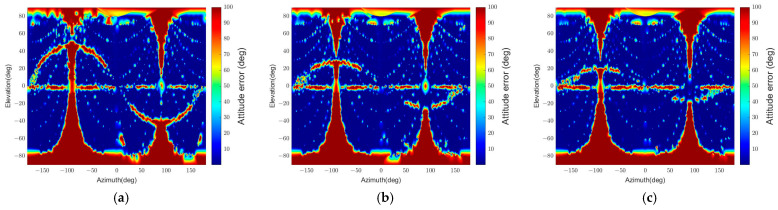
Relative attitude errors with variable size solar panels ($\left|r\right|$ = 30 m): (**a**) $W_{\mathrm{bp}}=1$, (**b**) $W_{\mathrm{bp}}=2$, (**c**) $W_{\mathrm{bp}}=3$.

The standard spacecraft model has a width ratio of 2, and the other two test cases have a width ratio of 1 and 3. Figure 17 describes the position error, and Figure 18 describes the attitude error for the three width ratios at a fixed relative distance. The most distinctive difference between the results in these figures is the elevation range of the sinusoidal failure line, marked as ‘D’ in Figure 15. The short-panel case has a higher maximum elevation than the long-panel case: specific values are 46.5°, 28°, and 20° for each.

The statistical results in Table 7 indicate no significant difference in pose initialization performance. The pass rate difference between the cases is less than 1.12%, and the outlier ratio difference is less than 0.66%. Therefore, we can use this algorithm even though the length of the panel is varied.

Second, the pose initialization algorithm is examined using images of the spacecraft with two and four panels, as shown in Figure 10. Due to symmetry along the ${\hat{b}}_{3}$ axis, some attitudes generate the same image. Unless the model embodies a recognizable structure that breaks the symmetry, the pose initialization algorithm cannot determine the attitude.

Although the shape of the target spacecraft is changed, the algorithm can estimate the pose of these spacecraft because we can observe the convexity defect on the contour between the panel and the body. Thus, the same 2D and 3D point combinations in Table 1 are used for model matching. The only difference is that we need to match and compare more cases than we do with the standard model because more than two convexity defects can be detected from these spacecraft. Accordingly, the number of feature point combinations increases. To improve the pose determination accuracy for symmetric spacecraft with four panels, we also considered convexity defects detected between panels. This convexity defect is not observed in the other two cases. Accordingly, the search space increases more than in the other two cases, which is checked through the increased execution time, and Table 8 shows the results, averaging the five repetitive execution times.

**Figure 19:**
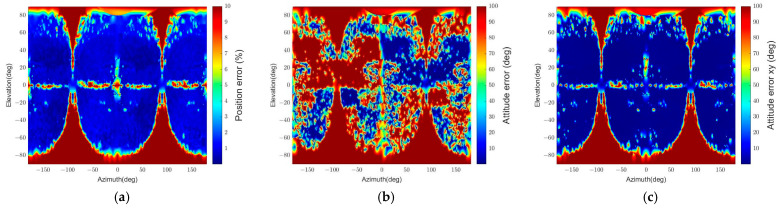
The relative position and attitude errors with the 2-panel spacecraft model ($\left|r\right|$ = 30 m): (**a**) relative position error, (**b**) relative attitude error, (**c**) relative attitude error of the ${\hat{b}}_{3}$ axis.

**Figure 20:**
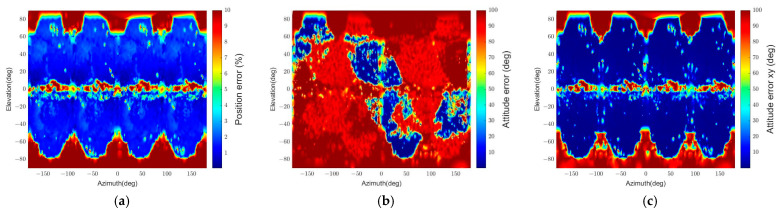
The relative position and attitude errors with the 4-panel spacecraft model ($\left|r\right|$ = 30 m): (**a**) relative position error, (**b**) relative attitude error, (**c**) relative attitude error of the ${\hat{b}}_{3}$ axis.

Figure 19 and Figure 20 depict the pose determination accuracy depending on the number of panels. The most distinctive change is that both figures’ relative attitude determination results described in (b) fail in most regions. This change occurs due to the symmetry along the ${\hat{b}}_{3}$ axis. On the other hand, if we investigate pose determination errors along the ${\hat{b}}_{1}$ and ${\hat{b}}_{2}$ axes, the relative attitude errors are significantly decreased, and their distribution is similar to the position error distribution, as shown in (a) and (c). These results indicate that the pose initialization algorithm can find a three degrees of freedom relative position and a two degrees of freedom relative attitude when applied to a symmetric spacecraft.

**Figure 21:**
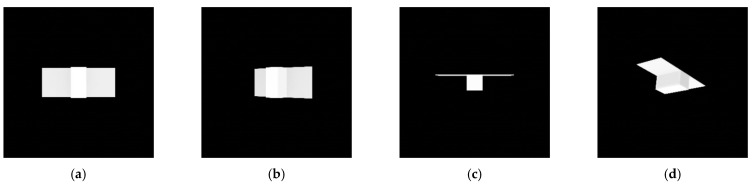
Example images for failure sections of 2-panel spacecraft: (**a**) failure section A, (**b**) failure section B, (**c**) failure section C, (**d**) failure section D.

**Figure 22:**
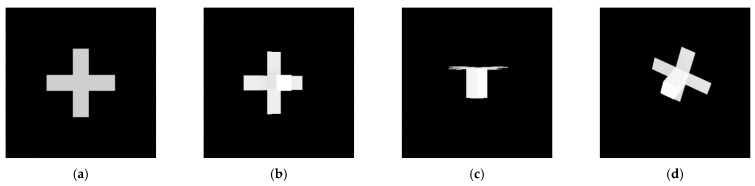
Example images for failure sections of 4-panel spacecraft: (**a**) failure section A, (**b**) failure section B, (**c**) failure section C, (**d**) failure section D.

The pose determination failure regions in 2-panel and 4-panel cases can also be categorized into four parts, as in the standard spacecraft model’s result in Figure 15. Figure 21 and Figure 22 represent the images of the target spacecraft from the pose determination failure parts. The 2-panel case has the same failure parts as the 1-panel case except for part D because more than one convexity defect can be observed in this shape, and when one panel fails to provide a convexity defect, the other panel can generate it. Similarly, the 4-panel case also has failure parts corresponding to parts A, B, and C, as shown in Figure 20. In this case, part D is not observed, and the areas in part B with elevations from approximately 10° to 60° and −10° to −60° have smaller errors. It is because other panels provide valid convexity defects, which is for the same reason as in the 2-panel case.

#### 5.3.3. Pose Estimation Performance with Textured-Surface Spacecraft

The final scenario uses textured spacecraft to quantify the performance degradation due to the light conditions. The textured model in this research includes a solar panel with black gloss on each side and a body covered with MLI, as shown in Figure 10. These textures give high contrast to images depending on the arrangement of the light source, the model, and the camera. The texture of the solar panel is designed to have zero transparency and a roughness of 0.4 using the principled BSDF shader in the 3D software Blender, and the texture of MLI is obtained from the IceSat2 model provided on the NASA 3D Resources website [63].

**Figure 23:**
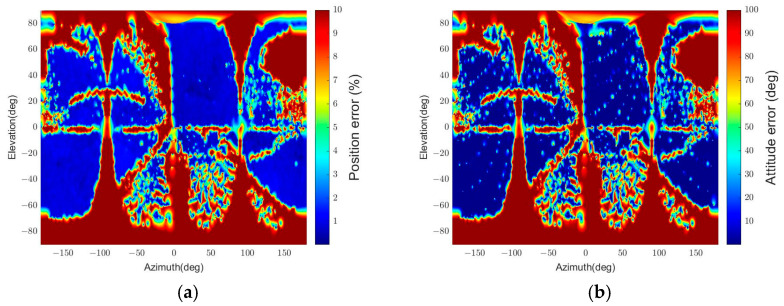
The relative position and attitude errors with the textured-surface spacecraft model ($\left|r\right|$ = 30 m): (**a**) relative position error, (**b**) relative attitude error.

The pose determination error is shown in Figure 23. The results indicate that the spacecraft’s surface characteristic decreases the pose estimation accuracy. In particular, the relative attitudes about −60° to 60° of azimuth and about 0° to −90° of elevation have noticeable degradation in the pose determination accuracy. The pass rate also drops because of the degradation, as Table 9 suggests. The cause of these results is that the feature detector cannot distinguish the solar panel and the spacecraft’s body from the image’s background when the sunlight does not reach them. On top of that, the rough surface of the spacecraft, covered with MLI, causes undesired extraction of MLI patterns instead of the spacecraft’s contour in image processing. Table 9 also describes the outlier ratios of this scenario, which increase from the ratios in the first scenario. However, the increase of the outlier ratios is less than 1%, which suggests that the pose initialization algorithm correctly filters out the wrong pose solutions.

In summary, the pose determination accuracy decreases due to the surface material’s reflectance and roughness. In contrast, the pose initialization algorithm can verify the correct poses to finish the initialization process.

## 6. Conclusions

This paper proposed a pose initialization algorithm to determine the relative pose of an uncooperative spacecraft without prior pose information. This algorithm was developed for a chaser spacecraft employing a monocular camera. Based on the assumption that the chaser spacecraft knows the model of a target spacecraft, the relative pose from the target to the chaser is computed using a convexity defect as a visual cue for finding 3D–2D point correspondences. The algorithm determines a relative pose from an image and finishes pose initialization when the pose has a small sum of reprojection errors. 

A preliminary simulation demonstrated the effectiveness of the designed model matching algorithm and the structure of the algorithm. Then, the pose determination accuracy of the algorithm was tested with five relative distances and all attitudes. The error increased when the distance between the spacecraft was extremely short or extremely far. Furthermore, pose determination failed when a convexity defect was not detected. In the second test scenario, the algorithm correctly estimated the relative pose of other spacecraft models in which the panel’s length or the number of panels was modified from the standard spacecraft model. The panel length did not affect the pose estimation accuracy. However, the increased number of panels caused symmetry in a spacecraft model, and the algorithm failed to estimate the symmetric model’s attitude accurately. Instead, attitudes along the other two asymmetrical axes were initialized with less than 2.1% of outliers. This two degrees of freedom attitude can be used to obtain the target’s pointing direction and capture the target with a robotic manipulator. 

The proposed algorithm has three major weaknesses that will be improved in future work. First, the algorithm is sensitive to illumination change, as we can check from the third test scenario. To improve the accuracy under harsh illumination conditions in the space environment, we can design a more robust image processing step to distinguish the spacecraft body from the background or use CNNs for robust feature detection. Second, the proposed algorithm cannot determine a pose if the contour in an image is convex. In order to determine a pose in this situation, we can also consider possible 3D–2D point correspondences when the contour is convex. Third, the algorithm was assessed with simplified spacecraft models. In real situations, a target spacecraft might have a more complex structure, and the algorithm will need an additional preprocessing step to simplify the target’s contour detected in an image. 

## Figures and Tables

**Table 1:** 2D and 3D point combinations in two cases and the number of iterations to test correspondences.

	Case 1	Case 2
2D point combinations	$\left(p_{1},\;p_{2},p_{3}\right)$	$\left(p_{1},\;p_{2,1},p_{3}\right)$ $\;\mathrm{or}\;\left(p_{1},\;p_{2,2},p_{3}\right)$
3D point combinations	$\left\{x_{1,}x_{6,}x_{7}\right\},\;\left\{x_{7,}x_{10,}x_{1}\right\},\left\{x_{6,}x_{1,}x_{10}\right\},\;\left\{x_{10,}x_{7,}x_{6}\right\},\left\{x_{7,}x_{6,}x_{1}\right\},\;\left\{x_{1,}x_{10,}x_{7}\right\},\;\left\{x_{10,}x_{1,}x_{6}\right\},\;\left\{x_{6,}x_{7,}x_{10}\right\}$	$\left\{x_{1},x_{6},x_{10}\right\},\left\{x_{6},x_{1},x_{7}\right\},\left\{x_{1},x_{3},x_{10}\right\},\;\left\{x_{6},x_{4},x_{7}\right\},\left\{x_{7},x_{10},x_{6}\right\},\left\{x_{10},x_{7},x_{1}\right\},\;\left\{x_{10},x_{6},x_{1}\right\},\left\{x_{7},x_{1},x_{6}\right\},\left\{x_{10},x_{3},x_{1}\right\},\;\left\{x_{7},x_{4},x_{6}\right\},\left\{x_{6},x_{10},x_{7}\right\},\left\{x_{1},x_{7},x_{10}\right\}$
Number of iterations	8	2 × 12 = 24

**Table 2:** Camera specification used in simulations.

Pixel Array Size	Focal Length	Pixel Size	Field of View
2048 × 2048	30 mm	7.4 μm× 7.4 μm	14°

**Table 3:** Dimensional properties of a standard spacecraft model.

Body	Solar Panel
3 m × 1.5 m × 1.5 m	3 m × 4.5 m × 0.05 m

**Table 4:** Execution time, pass rate, outlier ratio, and error statistics for different algorithms.

Algorithm	Time (s)	Pass Rate (%)	Outlier Ratio (%)	Relative Position		Relative Attitude	
				μ (%)	1σ (%)	μ (°)	1σ (°)
CDA	5449	80.86	2.37	0.96	0.22	0.68	0.38
RANSAC	303,963	86.06	3.27	0.94	0.32	0.78	0.57
CDA-simple	4696	80.75	2.45	0.97	0.42	0.83	0.48

**Table 5:** Pass rate and outlier ratio with different apparent angular sizes.

Apparent Angular Size	47.1°	23.5°	15.7°	9.4°	6.3°
Pass rate (%)	6.91	84.36	80.86	66.86	46.10
Outlier ratio (%)	4.25	2.23	2.37	2.46	3.57

**Table 6:** Pose initialization error statistics without outliers.

Apparent Angular Size		47.1°	23.5°	15.7°	9.4°	6.3°
Relative position	μ (%)	0.94	0.89	0.96	1.12	1.32
	1σ (%)	0.30	0.19	0.22	0.27	0.34
Relative attitude	μ (°)	0.55	0.62	0.68	0.85	1.10
	1σ (°)	0.60	0.37	0.38	0.45	0.57

**Table 7:** Pass rate and outlier ratio with the variable size of solar panels ($\left|r\right|$ = 30 m).

$W_{bp}$	1	2	3
Pass rate (%)	79.74	80.86	79.77
Outlier ratio (%)	2.87	2.37	2.21

**Table 8:** Computational time, pass rate, and outlier ratio for symmetric spacecraft ($\left|r\right|$ = 30 m).

No. Panels	1	2	4
Total execution time (s)	5449	6477	9666
Pass rate (%)	80.86	73.76	68.89
Outlier ratio (%)	0.34	1.39	2.02

**Table 9:** Pass rate and outlier ratio for the textured-surface spacecraft model.

Apparent Angular Size	23.5°	15.7°	9.4°
Pass rate (%)	51.41	56.04	50.63
Outlier ratio (%)	2.31	2.54	2.64

## Data Availability

Not applicable.

## References

[1] Cassinis L.P., Fonod R., Gill E. (2019). Review of the robustness and applicability of monocular pose estimation systems for relative navigation with an uncooperative spacecraft. Prog. Aerosp. Sci..

[2] Philip N., Ananthasayanam M., Dasgupta S. (1998). Study of Relative Position and Attitude Estimation and Control Scheme for the Final Phase of an Autonomous Docking Mission. IFAC Proc. Vol..

[3] Calhoun P.C., Dabney R. Solution to the problem of determining the relative 6 DOF state for spacecraft automated rendezvous and docking. Proceedings of the Space Guidance, Control, and Tracking II.

[4] Opromolla R., Fasano G., Rufino G., Grassi M. (2017). A review of cooperative and uncooperative spacecraft pose determination techniques for close-proximity operations. Prog. Aerosp. Sci..

[5] Kroes R., Montenbruck O., Bertiger W., Visser P. (2005). Precise GRACE baseline determination using GPS. Gps Solut..

[6] Gill E., D’Amico S., Montenbruck O. (2007). Autonomous formation flying for the PRISMA mission. J. Spacecr. Rocket..

[7] Kahr E., Roth N., Montenbruck O., Risi B., Zee R.E. (2018). GPS relative navigation for the CanX-4 and CanX-5 formation-flying nanosatellites. J. Spacecr. Rocket..

[8] Montenbruck O., Wermuth M., Kahle R. (2011). GPS based relative navigation for the TanDEM-X mission-first flight results. Navigation.

[9] Sarker S., Al-Tabatabai K.F., Pal A., Dhasarathan V., Arefin M.A., Islam M.K. (2021). D-shape photonic crystal fiber for optical coherence tomography: Design and analysis. Opt. Eng..

[10] Sarker S., Arefin M.A., Islam M.K. Design and FEM Analysis of a Novel Steering Shaped Photonic Crystal Fiber. Proceedings of the 2021 5th International Conference on Electrical Information and Communication Technology (EICT).

[11] Sarker S., Arefin M.A., Akram M.R., Islam M.K. High Nonlinearity and Ultra High Birefringence Silicon Core Photonic Crystal Fiber. Proceedings of the 2021 IEEE International Conference on Telecommunications and Photonics (ICTP).

[12] Di Mauro G., Lawn M., Bevilacqua R. (2018). Survey on guidance navigation and control requirements for spacecraft formation-flying missions. J. Guid. Control. Dyn..

[13] Ho C.-C.J., McClamroch N.H. (1993). Automatic spacecraft docking using computer vision-based guidance and control techniques. J. Guid. Control. Dyn..

[14] Junkins J.L., Hughes D.C., Wazni K.P., Pariyapong V. Vision-based navigation for rendezvous, docking and proximity operations. Proceedings of the 22nd Annual AAS Guidance and Control Conference.

[15] Sellmaier F., Boge T., Spurmann J., Gully S., Rupp T., Huber F. On-orbit servicing missions: Challenges and solutions for spacecraft operations. Proceedings of the SpaceOps 2010 Conference Delivering on the Dream Hosted by NASA Marshall Space Flight Center and Organized by AIAA.

[16] Davis J.P., Mayberry J.P., Penn J.P. (2019). On-orbit servicing: Inspection repair refuel upgrade and assembly of satellites in space. Aerosp. Corp. Rep..

[17] Nishida S.-I., Kawamoto S., Okawa Y., Terui F., Kitamura S. (2009). Space debris removal system using a small satellite. Acta Astronaut..

[18] D’Amico S., Benn M., Jørgensen J.L. (2014). Pose estimation of an uncooperative spacecraft from actual space imagery. Int. J. Space Sci. Eng. 5.

[19] Segal S., Carmi A., Gurfil P. (2013). Stereovision-based estimation of relative dynamics between noncooperative satellites: Theory and experiments. IEEE Trans. Control. Syst. Technol..

[20] Pesce V., Opromolla R., Sarno S., Lavagna M., Grassi M. (2019). Autonomous relative navigation around uncooperative spacecraft based on a single camera. Aerosp. Sci. Technol..

[21] Kelsey J.M., Byrne J., Cosgrove M., Seereeram S., Mehra R.K. Vision-based relative pose estimation for autonomous rendezvous and docking. Proceedings of the 2006 IEEE Aerospace Conference.

[22] Sharma S., Ventura J., D’Amico S. (2018). Robust model-based monocular pose initialization for noncooperative spacecraft rendezvous. J. Spacecr. Rocket..

[23] Chen Z., Li L., Wu Y., Hua B., Niu K. (2018). A new pose estimation method for non-cooperative spacecraft based on point cloud. Int. J. Intell. Comput. Cybern..

[24] Capuano V., Kim K., Harvard A., Chung S.-J. (2020). Monocular-based pose determination of uncooperative space objects. Acta Astronaut..

[25] Capuano V., Alimo S.R., Ho A.Q., Chung S.-J. Robust features extraction for on-board monocular-based spacecraft pose acquisition. Proceedings of the AIAA Scitech 2019 Forum.

[26] Shi J., Ulrich S., Ruel S. Spacecraft pose estimation using a monocular camera. Proceedings of the 67th International Astronautical Congress.

[27] Rondao D., Aouf N. Multi-view monocular pose estimation for spacecraft relative navigation. Proceedings of the 2018 AIAA Guidance, Navigation, and Control Conference.

[28] Hough P.V. (1962). Method and Means for Recognizing Complex Patterns. U.S. Patent.

[29] Alimohammadi S., He D. Multi-stage algorithm for uncertainty analysis of solar power forecasting. Proceedings of the 2016 IEEE Power and Energy Society General Meeting (PESGM).

[30] Von Gioi R.G., Jakubowicz J., Morel J.-M., Randall G. (2008). LSD: A fast line segment detector with a false detection control. IEEE Trans. Pattern Anal. Mach. Intell..

[31] Shi J. Good features to track. Proceedings of the 1994 IEEE Conference on Computer Vision and Pattern Recognition.

[32] Fischler M.A., Bolles R.C. (1981). Random sample consensus: A paradigm for model fitting with applications to image analysis and automated cartography. Commun. ACM.

[33] Abdi H., Williams L.J. (2010). Principal component analysis. Wiley Interdiscip. Rev. Comput. Stat..

[34] Harvard A., Capuano V., Shao E.Y., Chung S.-J. Spacecraft pose estimation from monocular images using neural network based keypoints and visibility maps. Proceedings of the AIAA Scitech 2020 Forum.

[35] Pasqualetto Cassinis L., Fonod R., Gill E., Ahrns I., Gil Fernandez J. CNN-based pose estimation system for close-proximity operations around uncooperative spacecraft. Proceedings of the AIAA Scitech 2020 Forum.

[36] Cassinis L.P., Fonod R., Gill E., Ahrns I., Gil-Fernández J. (2021). Evaluation of tightly-and loosely-coupled approaches in CNN-based pose estimation systems for uncooperative spacecraft. Acta Astronaut..

[37] Szeliski R. (2011). Feature-based alignment. Computer Vision.

[38] Gao X.-S., Hou X.-R., Tang J., Cheng H.-F. (2003). Complete solution classification for the perspective-three-point problem. IEEE Trans. Pattern Anal. Mach. Intell..

[39] Bujnak M., Kukelova Z., Pajdla T. A general solution to the P4P problem for camera with unknown focal length. Proceedings of the 2008 IEEE Conference on Computer Vision and Pattern Recognition.

[40] Lepetit V., Moreno-Noguer F., Fua P. (2009). Epnp: An accurate o (n) solution to the pnp problem. Int. J. Comput. Vis..

[41] Pesce V., Haydar M.F., Lavagna M., Lovera M. (2019). Comparison of filtering techniques for relative attitude estimation of uncooperative space objects. Aerosp. Sci. Technol..

[42] David P., Dementhon D., Duraiswami R., Samet H. (2004). SoftPOSIT: Simultaneous pose and correspondence determination. Int. J. Comput. Vis..

[43] Sharma S., Beierle C., D’Amico S. Pose estimation for non-cooperative spacecraft rendezvous using convolutional neural networks. Proceedings of the 2018 IEEE Aerospace Conference.

[44] Kalchbrenner N., Grefenstette E., Blunsom P. (2014). A convolutional neural network for modelling sentences. arXiv.

[45] Cassinis L.P., Menicucci A., Gill E., Ahrns I., Sanchez-Gestido M. (2022). On-ground validation of a CNN-based monocular pose estimation system for uncooperative spacecraft: Bridging domain shift in rendezvous scenarios. Acta Astronaut..

[46] Park T.H., D’Amico S. (2022). Robust Multi-Task Learning and Online Refinement for Spacecraft Pose Estimation across Domain Gap. arXiv.

[47] Park T.H., Märtens M., Lecuyer G., Izzo D., D’Amico S. (2021). SPEED+: Next Generation Dataset for Spacecraft Pose Estimation across Domain Gap. arXiv.

[48] Suzuki S. (1985). Topological structural analysis of digitized binary images by border following. Comput. Vis. Graph. Image Processing.

[49] Saalfeld A. (1999). Topologically consistent line simplification with the Douglas-Peucker algorithm. Cartogr. Geogr. Inf. Sci..

[50] Fiorenza C.E., Barik S.K., Prajapati A., Mahesh S. (2019). Hand Gesture Recognition using Convexity Defects. Int. J. Innov. Technol. Explor. Eng. (IJITEE).

[51] Boyd S., Boyd S.P., Vandenberghe L. (2004). Convex Optimization.

[52] Contours: More functions. https://docs.opencv.org/3.4/d8/d1c/tutorial_js_contours_more_functions.html.

[53] Graham R.L. (1972). An efficient algorithm for determining the convex hull of a finite planar set. Info. Pro. Lett..

[54] Jarvis R.A. (1973). On the identification of the convex hull of a finite set of points in the plane. Inf. Processing Lett..

[55] Bradski G. (2000). The OpenCV Library. Dr. Dobb’s J. Softw. Tools.

[56] Rezatofighi H., Tsoi N., Gwak J., Sadeghian A., Reid I., Savarese S. Generalized intersection over union: A metric and a loss for bounding box regression. Proceedings of the IEEE/CVF Conference on Computer Vision and Pattern Recognition.

[57] Community B.O. (2018). Blender—A 3D Modelling and Rendering Package.

[58] Capuano G., Severi M., Cacace F., Lirato R., Longobardi P., Pollio G., DeNino M., Ippolito M. Video system for prisma formation flying mission. Proceedings of the IAA Symposium on Small Satellite Systems and Services (4S).

[59] Woffinden D.C., Geller D.K. (2007). Relative angles-only navigation and pose estimation for autonomous orbital rendezvous. J. Guid. Control. Dyn..

[60] Sharma S. (2016). Comparative assessment of techniques for initial pose estimation using monocular vision. Acta Astronaut..

[61] Guo K., Ye H., Gao X., Chen H. (2022). An Accurate and Robust Method for Absolute Pose Estimation with UAV Using RANSAC. Sensors.

[62] Li S., Xu C., Xie M. (2012). A robust O (n) solution to the perspective-n-point problem. IEEE Trans. Pattern Anal. Mach. Intell..

[63] Kumanchik B. ICESAT2. https://nasa3d.arc.nasa.gov/detail/jpl-IceSat2.

